# Tissue-resident memory T cells in renal autoimmune diseases

**DOI:** 10.3389/fimmu.2023.1111521

**Published:** 2023-01-23

**Authors:** Pauline Ginsberg, Ulf Panzer, Nariaki Asada

**Affiliations:** III. Department of Medicine, Division of Translational Immunology, University Medical Hospital Center Hamburg-Eppendorf, Hamburg, Germany

**Keywords:** tissue-resident memory T cell, renal autoimmune disease, crescentic glomerulonephritis, lupus nephritis, ANCA-associated glomerulonephritis

## Abstract

The discovery of tissue-resident memory T cells (T_RM_ cells) reinterpreted the potential of human tissue-specific immunity. Following T cell receptor (TCR) activation and clonal expansion, effector T cells migrate to peripheral tissues where they remain long-term and differentiate to T_RM_ cells after antigen clearance. This allows for prompt immunological responses upon antigen re-encounter. In addition to their protective properties in acute infections, recent studies have revealed that T_RM_ cells might lead to aggravation of autoimmune diseases, such as lupus nephritis (LN) and anti-neutrophil cytoplasmic antibody (ANCA)-associated glomerulonephritis (GN). These diseases present as proliferative and crescentic glomerulonephritis (cGN), which is a life-threatening condition leading to end-stage renal disease (ESRD) if left untreated. A better understanding of renal T_RM_ cells might lead to identifying new therapeutic targets for relapsing autoimmune diseases of the kidney. In this review, we summarize the current knowledge of renal T_RM_ cells and discuss their potential pathophysiological roles in renal autoimmune diseases.

## Introduction

Tissue-resident memory T cells (T_RM_ cells) are a recently identified lymphocyte subset that resides in peripheral organs for long time periods (1, 2). These cells have been detected and analyzed in numerous non-lymphoid tissues, especially barrier-organs. Mice studies have proven that T_RM_ cells have superior effector functions including rapid chemokine and cytokine production. Hence, together with circulating memory T cells (e.g., effector memory T cells and central memory T cells), T_RM_ cells are critical for efficient pathogen clearance, providing an antigen-specific immediate frontline defense in peripheral organs (1, 2).

**Figure 1:**
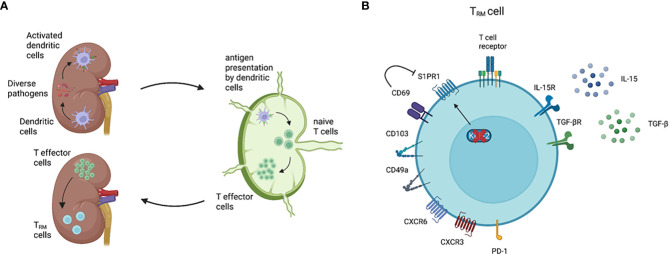
General features of T_RM_ cells. Renal T_RM_ cell development and phenotype. **(A)** As classical antigen-presenting cells (APC), dendritic cells (DCs) constantly surveil their microenvironment for pathogens and microbes. Once DCs in the kidney are activated, these cells migrate to the renal draining lymph nodes to present the processed antigen *via* the major histocompatibility complex (MHC) to naïve T cells (3). Upon activation, naïve T cells become effector T cells and migrate to the inflamed kidney *via* a chemokine gradient (4). While most of the effector T cells undergo apoptosis following the resolution of inflammation, a small subset of the effector T cells become T_RM_ cells. T_RM_ cells are the most abundant immune cell population in the healthy human kidney (5). **(B)** A T_RM_ cell marker CD69 downregulates the expression of S1PR1, which is a receptor required for tissue egress. The expression of transcription factor KLF-2, which upregulates S1PR1 expression, is downregulated in T_RM_ cells (6). CD103 and CD49a are parts of the integrins αEβ7 and α1β1, respectively. CD103 interacts with E-cadherin on epithelial cells, whereas CD49a binds to collagen IV (2, 7). Almost all renal T_RM_ cells express the chemokine receptor CXCR3 and, to a lower degree, CXCR6 (8). Inhibitory receptors, especially PD-1, are commonly expressed on T_RM_ cells compared with circulating memory T cells. IL-15 and TGF-β are required to induce and maintain renal T_RM_ cells in mice (9, 10). Created with BioRender.com

The kidney is a non-barrier organ, but still frequently gets challenged by diverse pathogens, including bacteria, viruses, and fungi, which drive renal T_RM_ cell generation (**Figure 1A**) (2, 5, 11–14). Due to limited access, exploring precise immune cell populations of healthy human kidneys had been challenging. In the last few years, however, detailed analysis of the immune landscape in healthy renal tissue has been implemented using flow cytometry and single-cell RNA sequencing (scRNA-seq) (2, 5, 8, 15–17). In particular, the combined analysis of mRNA expression and cell surface marker *via* cellular indexing of transcriptomes and epitopes by sequencing (CITE-seq) has provided a completely new insight into cell heterogeneity. Consequently, CD4^+^ and CD8^+^ T cells with a T_RM_ cell phenotype were identified in the healthy human kidney, representing the most abundant tissue-resident immune cell population (8, 16, 17).

Glomerulonephritis (GN) is a group of immune-mediated kidney diseases, either in the context of systemic or kidney-specific autoimmune disorders, and lead to the damage of the glomeruli and other compartments of the kidney (18). The most severe form is crescentic GN (cGN) or rapid-progressive glomerulonephritis (RPGN), which is mainly observed in autoimmune kidney diseases such as lupus nephritis (LN) and ANCA-associated GN. The histopathological features of cGN are multilayered cells in the Bowman’s capsule due to epithelial hyperplasia (crescents) and infiltration of various immune cells in the periglomerular area (19). Importantly, T cells are one of the most abundant immune cell populations in the inflamed kidney tissue. Most of the renal T cells have a tissue-resident phenotype (17, 20, 21), and the number of tissue-resident T cells correlates with disease severity (17, 22–25). Moreover, recent studies reported that T_RM_ cells might be associated with the development and/or relapse of autoimmune diseases (2, 17, 26). For these reasons, it is of great importance to summarize and discuss the current understanding of renal T_RM_ cells. From the next section, we will shortly explain the current knowledge of T_RM_ cells and then discuss the role of T_RM_ cells in renal autoimmune diseases.

## General features of T_RM_ cells

T_RM_ cells are particularly characterized by two features – tissue residency and longevity – which are identified by distinct surface markers and transcriptional profiles (2, 14, 27). Similar to other non-lymphoid organs, tissue-residency of renal T_RM_ cells is mainly assessed by the expression of CD69 and resident-integrins such as CD103 and CD49a, and the downregulation of transcription factor KLF-2, all of which contribute to preventing tissue egress of T_RM_ cells (**Figure 1B**) (2, 6, 7, 14, 28). The longevity of T_RM_ cells is mainly regulated by a transcriptional network which enables adaptation to a certain tissue environment with a constant supply of cytokines and nutrients. Mice studies showed that IL-15 and TGF-β are important for the development and survival of renal T_RM_ cells after viral infection (9, 10, 29). Regarding nutrition, T_RM_ cells in the skin utilize exogenous free fatty acids (FFA) for oxidative phosphorylation (OXPHOS) to generate ATP (30). Given that the human kidney is a highly hypoxic organ, which might inhibit efficient OXPHOS (31), further tissue-specific analysis is crucial to determine the FFA-dependency of renal T_RM_ cells.

T_RM_ cells can be both protective and pathogenic. In the skin, pathogen- or sterile inflammation-triggered T_RM_ cells show protective properties leading to enhanced pathogen clearance upon antigen re-encounter (32, 33). On the other hand, auto-antigen specific T_RM_ cells can be constantly exposed to self-antigens, contributing to chronic inflammation in autoimmune skin diseases (34). Because of the emerging pathophysiological role in relapsing autoimmune skin diseases, T_RM_ cells have been attracting attention in the field of nephrology as well (2, 17, 26). In human kidneys, the presence of T_RM_ cells specific to systemic virus infections (Epstein-Barr virus, cytomegalovirus, influenza A, and BK virus) has been reported (2, 5, 14), although their functional relevance such as accelerated host defense still needs to be clarified.

## T_RM_ cells in lupus nephritis

Systemic lupus erythematosus (SLE) is a systemic and chronic autoimmune disease, which typically manifests in several organs and tissues. The etiology of SLE is still not fully understood, but genetic susceptibility, environmental influences, hormones, and dysregulation of the innate and adaptive immune system have been implicated (35, 36). In SLE, up to 50% of patients develop LN (37). The main pathophysiological driver of renal tissue injury is the loss of self-tolerance, which leads to the production of autoantibodies against nuclear antigens and the accumulation of immune complexes within the glomeruli (38). These immune complexes activate the complement system, inducing a massive tissue infiltration of immune cells.

T cells are the most abundant immune cells in biopsies of patients suffering from LN as well as in lupus-prone mice kidneys (21, 39, 40). T cells localize mainly in the interstitial and periglomerular regions in LN biopsies (39–41). Depletion of infiltrating T cells by antibody injection into MRL/lpr mice significantly reduced renal inflammation and tissue damage, underlining the pathophysiologic role of T cells in LN. Furthermore, T cell infiltration correlates with clinical parameters such as proteinuria, serum creatinine levels, and histopathological grading (22–25).

Flow cytometric analyses of human LN biopsies and MRL/lpr mice revealed that most of the kidney-infiltrating T cells have T_RM_ cell phenotype, such as the expression of CD69 and/or CD103. In addition, the numbers of CD4^+^ and CD8^+^ T_RM_ cells in LN kidney are significantly higher compared with healthy control tissue (20, 21). Recent studies using scRNA-seq determined the immune cell composition and transcription profiles of the kidney-infiltrating immune cells during LN (26, 42). The analysis of human LN biopsies revealed the presence of different T cell clusters in the kidney and showed that feature genes of CD8^+^ T_RM_ cells include *ZNF683* (HOBIT), *ITGAE*, *ITGA1*, and *XCL1* (26).

Renal T_RM_ cells in LN might have enhanced effector functions. CD8^+^ CD103^+^ T_RM_ cells from MRL/lpr mice kidneys showed the superior production of cytokines and cytotoxic molecules, such as TNF-α, IFN-γ, perforin, and granzyme B, in comparison to CD103^-^ non-T_RM_ cells (20). However, another study revealed contrasting results, showing an impaired cytokine production in association with the expression of inhibitory receptors, such as PD-1 and Tim3, which are generally observed during T cell exhaustion (21). A scRNA-seq analysis of the murine lupus model showed an exhaustion signature in both CD4^+^ and CD8^+^ renal T cells, suggesting that kidney-infiltrating T cells gradually develop T cell exhaustion (42). Trajectory analyses revealed that CD4^+^ and CD8^+^ T cells might go through several developmental stages. They first exhibit effector functions during migration to the tissue, and then upregulate hypoxia-inducible genes which finally lead to the T cell exhaustion (42, 43).

T cell clonality analysis is of great importance for better understanding adaptive immunity in health and disease. In autoimmune diseases, autoreactive T cells clonally expand and accumulate in inflamed organs (44–46). TCR ß-chain sequencing combined with laser-capture microdissection from kidney biopsies revealed the predominance of clonally expanded CD4^+^ and CD8^+^ T cells in periglomerular regions, suggesting both T cells play roles in the progression of LN (46). In addition, a recent study reported that the frequency of nuclear antigen-reactive CD4^+^ T cells correlated with disease severity (47). This study revealed the presence of CD4^+^ T cells specific to canonical nuclear antigens SmD1, RNP70, histone, Ro, and La in patients with LN.

During kidney inflammation, leukocytes including T cells can be found in the urine (48–50). Mass cytometry analysis of urinary T cells from LN patients revealed the expression of CD69, indicating that even T cells with a tissue-resident phenotype can be observed in the urine (50). Furthermore, the amount of T cells in the urine correlates with disease activity as well as severity, thus can be used as a biomarker in LN (49). Interestingly, nuclear antigen-reactive T cells are enriched in the urine of LN patients compared with the blood. This indicates that urinary T cells might, to some extent, reflect T cell clonality in the inflamed kidney tissue during LN (47).

## T_RM_ cells in ANCA-associated GN

ANCA-associated vasculitis (AAV) is a group of autoimmune diseases affecting small-sized vessels, encompassing microscopic polyangiitis (MPA), granulomatosis with polyangiitis (GPA), and eosinophilic granulomatosis with polyangiitis (EGPA) (51–53). Similar to SLE, the etiology of AAV is still not fully understood, but generally considered to be the result of multiple factors, including genetic susceptibility (e.g., MHC II genotypes) and environmental factors, such as pathogens and drugs (54–57). All AAV can affect the kidney and induce cGN. Each disease is commonly characterized by auto-antibodies directed against specific antigens, namely myeloperoxidase (MPO) and proteinase 3 (PR3). In general, MPO-ANCA is common in MPA, whereas PR3-ANCA is associated with GPA. Both antigens are proteins located within neutrophils and are unleashed from the cells following activation (53). Upon bacterial infections, neutrophils are activated by pro-inflammatory cytokines as well as direct contact with the pathogen, and initiate neutrophil extracellular traps (NETs) formation. Under physiological circumstances, NETs are cleared in a timely manner by specific enzymes, such as DNase I. In AAV patients, the degradation of NETs is impaired, leading to the persistent NET formation and disrupted tolerance to auto-antigens, such as MPO and PR3, resulting in the production of auto-antibodies MPO- and PR3-ANCA. ANCAs bind to neutrophils, leading to changes in adhesion molecule expression, the release of reactive oxygen species, cytokines, and lytic enzymes, and eventually induction of necrotizing vasculitis (58).

In ANCA-associated GN, the accumulation of T cells in the inflamed kidney tissue is observed (59). The number of CD4^+^ and CD8^+^ T cells correlates with kidney function. Furthermore, in the glomeruli with segmental necrosis and cellular crescents, active glomerular lesions correlate with intraglomerular T cells (59). In AAV patients, MPO- and PR3-specific CD4^+^ T cells were identified, showing a decreased TCR diversity and clonal expansion (55–57). These autoreactive T cells show memory T cell phenotype and secrete proinflammatory cytokines including IL-17A (55, 60).

A scRNA-seq analysis of T cells from ANCA-associated GN biopsies showed a variety of T cell populations in the inflamed kidney tissue. Those T cells include CD4^+^ T_RM_ cells with different transcriptome profiles, such as type1 or typ3 immunity-associated gene expression (17). The number of CD69^+^ T_RM_ cells was significantly higher in ANCA-associated GN biopsies compared to healthy renal tissue, indicating the recruitment of autoreactive T cells into the kidneys of patients (17). As observed in LN, the abundance of T_RM_ cells positively correlated with disease activity. The T_RM_ cells from ANCA-associated GN showed an upregulation of genes involved in T cell proliferation, activation, and cytokine signaling compared with healthy control (17).

While antigen-specific immunity is the central function of T cells, TCR-independent activation (bystander T cell activation) has also been reported (61). Upon activation by cytokines, bystander T cells can immediately secrete effector cytokines in the absence of antigen stimulation. This finding suggests that the induction of renal T_RM_ cells can be harmful to the kidney tissue regardless of the antigen-specificity. In mice studies, systemic infection of *Staphylococcus aureus* or *Candida albicans* induced renal CD4^+^ T_RM_ cells including T_RM_17 cells, which secrete IL-17A upon reactivation. The presence of T_RM_ cells significantly aggravated the progression of experimental GN, indicating that bystander T_RM_ cells contributed to GN independent of TCR signaling. *In vitro* study revealed that T_RM_ cells can be activated in the presence of a cytokine cocktail of IL-23, IL-6, and IL-1β (17). Therefore, future analysis should focus on not only autoreactive T cells but also activated bystander T cells in autoimmune kidney diseases.

## Treatment strategies for renal autoimmune diseases

After diagnosing kidney involvement in systemic autoimmune diseases such as SLE and AAV, therapeutic intervention is needed to prevent progressive kidney injury. In general, therapy can be divided into two phases – induction therapy to rapidly suppress inflammation and maintenance therapy to prevent relapse. Current treatment strategies are largely based on systemic immunosuppressants (**Table 1**) (62, 63).

**Table 1:** Simplified treatment strategies in LN and ANCA-associated GN (esp. active, severe GPA/MPA) (62, 63).

	LN	ANCA-associated GN
Induction therapy	Hydroxychloroquine (basic therapy) + Glucocorticoids + Mycophenolate mofetil + Cyclophosphamide	Glucocorticoids + Rituximab or Cyclophosphamide (Plasma exchange)
Maintenance therapy	Hydroxychloroquine (basic therapy) + Mycophenolate mofetil or Azathioprine	Rituximab or Azathioprine
Add-On	Renin-angiotensin-aldosterone system blockers Belimumab (SGLT2 inhibitors)	Renin-angiotensin-aldosterone system blockers Mycophenolate mofetil Avacopan (SGLT2 inhibitors)

Generally, the therapy regimen depends on disease severity and the risk of progression. Moreover, the therapy regimen changes according to the type of ANCA-associated GN.

Some therapeutic strategies for LN or ANCA-associated GN target B cells and/or plasma cells. The number of autoantibody-producing cells can be reduced by using rituximab, belimumab, or daratumumab, which are monoclonal antibodies against CD20, B-cell activating factor (BAFF), or CD38, respectively (63–65). In ANCA-associated GN, the complement pathway can also be targeted. Avacopan, an orally available C5a receptor antagonist which blocks neutrophil chemoattraction and activation, is noninferior to prednisone taper with respect to remission and shows better eGFR recovery (3). Despite the therapeutic progress, efficient treatment strategy targeting T cells has not been established.

Recent studies suggested that T_RM_ cells can be targeted by diverse strategies. Firstly, T_RM_ cell-specific metabolic pathways can be modulated. T_RM_ cells require exogenous FFA uptake for their survival and function, and T cell-specific deletion of FABP4/5 impairs the development and function of T_RM_ cells (30). Therefore, inhibiting FFA uptake can be an efficient treatment strategy targeting T_RM_ cells. However, FFA-dependency and general metabolic pathways of renal T_RM_ cells still need to be determined. Secondly, cytokines essential for long-term survival of T_RM_ cells, e.g., IL-15, can be targeted to reduce the cells (66). CD122, which is a subunit of IL-15 and IL-2 receptor, is expressed by T_RM_ cells as well as effector T cells (67). In vitiligo, an autoimmune disease affecting the skin, the number of T_RM_ cells was successfully reduced by an anti-CD122 antibody therapy (67). Short-term treatment with the anti-CD122 therapy inhibited the production of cytokine by T_RM_ cells, and long-term treatment depleted T_RM_ cells from skin lesions. Given CD122 is expressed by T_RM_ cells in different organs, the anti-CD122 therapy might be effective for renal T_RM_ cells as well. Moreover, downstream of IL-15 and CD122, such as JAK-STAT pathways, can be blocked. Tofacitinib, an inhibitor of JAK1 and JAK3, impaired the survival of renal T_RM_ cells, contributing to improved kidney function in MRL/lpr lupus mice model (20). Thirdly, autoreactive T cell-specific intervention might be possible. Diverse methods are currently under investigation to induce antigen-specific immune tolerance, and the methods include autoantigen-coated microparticles, autoantigen peptide-MHC multimers, and antigen-specific regulatory T cell transfer (68). In murine models of type I diabetes, multiple sclerosis and arthritis, disease-relevant peptide-MHC class II-nanoparticles successfully reversed established disease in association with systemic expansions of cognate CD4^+^ T cells displaying a type 1 regulatory T cell phenotype (68). Importantly, these peptide-MHC-based nanomedicines could suppress autoimmune diseases without suppressing systemic immunity. In order to develop T_RM_ cell-targeting therapeutic strategies, further studies should focus on characterizing renal T_RM_ cells and microenvironments in different disease conditions.

## Conclusion

The discovery of T_RM_ cells has significantly improved our understanding of immunology. Several studies analyzing animal models and patients with renal autoimmune diseases successfully identified CD4^+^ and CD8^+^ T_RM_ cell populations in the kidney and showed potential clinical relevance. Autoreactive T_RM_ cells can be clonally expanded due to self-antigen exposure, contributing to autoimmune kidney diseases (44–46). Of interest, T_RM_ cells induced by infections also stay in the kidney and can be activated during autoimmunity (bystander T cell activation) promoting tissue damage (17). Better characterization of T_RM_ cells and microenvironment in renal autoimmune diseases might lead to the development of T_RM_ cell-specific therapeutic strategies.

## Author contributions

PG wrote a draft. NA and UP supervised and finalized the manuscript. All authors contributed to the article and approved the submitted version.
